# Rapid evolution of fluoroquinolone-resistant *Escherichia coli *in Nigeria is temporally associated with fluoroquinolone use

**DOI:** 10.1186/1471-2334-11-312

**Published:** 2011-11-07

**Authors:** Adebayo Lamikanra, Jennifer L Crowe, Rebeccah S Lijek, Babatunde W Odetoyin, John Wain, A Oladipo Aboderin, Iruka N Okeke

**Affiliations:** 1 Faculty of Pharmacy, Obafemi Awolowo University, Ile-Ife, Osun State, Nigeria; 2 Department of Biology, Haverford College, 370 Lancaster Avenue, Haverford, PA 19041, USA; 3 Department of Medical Microbiology and Parasitology, Obafemi Awolowo University, Ile-Ife, Osun State Nigeria; 4 Health Protection Agency, Colindale, London, NW9 5EQ, UK

**Keywords:** antimicrobial resistance, antimicrobial use, quinolone resistance, drug resistance, ciprofloxacin, fluoroquinolones, selective pressure, Nigeria, chloroquine, antimalarial, fluoroquinolone-resistant, *Escherichia coli*

## Abstract

**Background:**

Antibiotic resistance has necessitated fluoroquinolone use but little is known about the selective forces and resistance trajectory in malaria-endemic settings, where selection from the antimalarial chloroquine for fluoroquinolone-resistant bacteria has been proposed.

**Methods:**

Antimicrobial resistance was studied in fecal *Escherichia coli *isolates in a Nigerian community. Quinolone-resistance determining regions of *gyrA *and *parC *were sequenced in nalidixic acid resistant strains and horizontally-transmitted quinolone-resistance genes were sought by PCR. Antimicrobial prescription practices were compared with antimicrobial resistance rates over a period spanning three decades.

**Results:**

Before 2005, quinolone resistance was limited to low-level nalixidic acid resistance in fewer than 4% of *E. coli *isolates. In 2005, the proportion of isolates demonstrating low-level quinolone resistance due to elevated efflux increased and high-level quinolone resistance and resistance to the fluoroquinolones appeared. Fluoroquinolone resistance was attributable to single nucleotide polymorphisms in quinolone target genes *gyrA *and/or *parC*. By 2009, 35 (34.5%) of isolates were quinolone non-susceptible with nine carrying *gyrA *and *parC *SNPs and six bearing identical *qnrS1 *alleles. The antimalarial chloroquine was heavily used throughout the entire period but *E. coli *with quinolone-specific resistance mechanisms were only detected in the final half decade, immediately following the introduction of the fluoroquinolone antibacterial ciprofloxacin.

**Conclusions:**

Fluoroquinolones, and not chloroquine, appear to be the selective force for fluoroquinolone-resistant fecal *E. coli *in this setting. Rapid evolution to resistance following fluoroquinolone introduction points the need to implement resistant containment strategies when new antibacterials are introduced into resource-poor settings with high infectious disease burdens.

## 1. Background

Chloroquine has been described as one of the most important drugs ever used to treat an infection. This cheap and effective drug was the primary antimalarial worldwide until resistance emerged in the 1970s [1]. Multiple alleles of a chloroquine resistance transporter gene, *pfcrt*, and a multidrug resistance transporter gene, *pfmdr1*, are associated with chloroquine resistance in *Plasmodium falciparum*. Resistant *P. falciparum *haplotypes that predominate in sub-Saharan Africa appear to possess a fitness disadvantage, suggesting that they are maintained by selective pressure from chloroquine, which is still heavily used [2,3]. Davidson et al [4] recently proposed that, in addition to the selective pressure for chloroquine-resistant plasmodia, chloroquine's weak antibacterial activity confers sufficient selective advantage for fluoroquinolone resistant *Escherichia coli *(FQREC), which emerged in rural Guyana in the absence of antibacterial quinolone use. If this cheap antimalarial selects for fluoroquinolone-resistant bacteria, justification for reintroduction-by combining it with resistance-reversing chemicals [5] or by withdrawing it until susceptibility is returned and then reintroducing it in combination with other antimalarials [2]-could decrease because of the great and growing need for fluoroquinolones. However, little is known about resistance, drug consumption and selection in malaria-endemic Africa, where high levels of chloroquine and fluoroquinolone use now occurs concurrently.

The quinolone antibacterial nalidixic acid was originally derived from a byproduct of chloroquine synthesis and shares structural features with chloroquine [6]. Nalidixic acid and its more active derivatives, the fluoroquinolones, were rarely used in Africa before the late 1990s because of their high cost. More recently, bacterial resistance to cheaper antibacterials has necessitated widespread fluoroquinolone use. Patents protecting ciprofloxacin and perfloxacin have recently expired, allowing for cheaper generics to be disseminated widely. Fluoroquinolones are stable, orally administrable, and now affordable. In the last decade, fluoroquinolones have become first- and second-line antibacterials of choice for acute respiratory, enteric and urinary tract infections as well as serious systemic infections such as bacteremia [7,8]. Fluoroquinolones are also employed in combination with other antimicrobials to treat multidrug resistant tuberculosis [9,10]. Additionally, their use and misuse in the informal sector, by unsanctioned providers and through self medication, is commonplace in Nigeria and in many other parts of Africa [11].

Quinolones inhibit the activity of bacterial DNA gyrase and topoisomerase enzymes, which are essential for replication [12]. Single nucleotide polymorphisms in the quinolone resistance determining regions (QRDR) of *gyrA *and *parC*, two of the genes that encode DNA gyrase and topoisomerase IV respectively, can lead to conformational changes in these enzymes that prevent quinolones from binding but still preserve their enzymatic function [13]. In *E. coli *and related Gram-negative bacteria, DNA gyrase is the first target for fluoroquinolones. If *gyrA *has resistance-conferring mutations, the primary target of fluoroquinolones switches from DNA gyrase to topoisomerase IV [12,14,15]. Mutations in the QRDRs of *gyrA *and *parC *are the most commonly documented quinolone resistance mechanisms, but resistance is also known to be conferred by mutations in the second topoisomerase gene, *parE *[14]. Quinolone resistance can also be acquired horizontally through transferable *aac(6*'*)-Ib *or quinolone resistance (*qnr*) DNA. AAC(6')-Ib encodes a ciprofloxacin acetylating enzyme, while the product of *qnr *inhibits quinolones binding to target proteins [16,17]. Another mechanism of quinolone resistance relies on upregulation of efflux pumps that export quinolones and other antimicrobials out of the bacterial cell. For example, mutations in the gene *acrR*, which encodes a repressor of the *acrAB *pump genes, are associated with quinolone resistance [18] Recently, *qepA *and *oqxAB*, which encode horizontally-transmitted efflux pumps have also been described [17,19].

Low-level resistance to the quinolones often evolves by acquisition of one resistance-conferring mutation or gene. Subsequent genetic changes lead to higher levels of resistance to the first generation quinolone nalidixic acid and a broadening of the resistance spectrum to include second-generation quinolones (first generation fluoroquinolones) such as ciprofloxacin, followed by newer second- and third-generation fluoroquinolones that are yet to reach markets in Nigeria and most other African countries [12,20]. Although multiple mechanisms of quinolone resistance have been reported from other continents, there are very few data from sub-Saharan Africa on the molecular basis for quinolone resistance. Prior to 2009, horizontally-acquired resistance had not been reported. If cross-selection from chloroquine results in antibacterial resistance more globally, then 1) FQREC should be commonplace in equatorial Africa, which has seen the greatest pressure from chloroquine, and 2) FQREC would have evolved before the introduction of quinolone antibacterials. We report quinolone susceptibility trends among commensal *E. coli *isolated from apparently healthy undergraduate students over three decades and antimicrobial use at the University health center over that period. Quinolone resistance emerged within the last decade and was coincident with the introduction of the fluoroquinolone ciprofloxacin.

## 2. Methods

### 2.1. Bacterial isolates

Permission to conduct this study was provided by the Institutional Review Board of Obafemi Awolowo University, Ile-Ife, Nigeria. *E. coli *isolates were recovered from stool specimens collected from apparently healthy undergraduates at Obafemi Awolowo University as described previously [21]. Data from isolates collected in 1986-1998 has been described previously. For this study, we collected and processed specimens in 2005 and 2009 using identical protocols. All volunteers submitting a stool specimen gave informed consent. The identity of colonies with a typical *E. coli *morphology on MacConkey agar was confirmed by biochemical testing. Colonies from the same specimen with identical biochemical and susceptibility profiles were treated as identical isolates.

### 2.2. Antimicrobial susceptibility testing

Each strain was tested for susceptibility to eight antimicrobials using the Clinical and Laboratory Standards Institute (CLSI, formerly NCCLS) disc diffusion method [22]. Discs used contained ampicillin (10 μg), streptomycin (10 μg), trimethoprim (5 μg), tetracycline (30 μg), nalidixic acid (30 μg), chloramphenicol (30 μg), ciprofloxacin (5 μg) and sulphonamide (300 μg) (Oxoid/Remel) and *E. coli *ATCC 35218 was used as control strain. Inhibition zone diameters were interpreted in accordance with CLSI guidelines using WHONET software version 5.3 [23]. Minimum inhibitory concentrations (MICs) to nalidixic acid were measured using the agar dilution technique on Mueller-Hinton agar as recommended by the CLSI and using *E. coli *ATCC 35218 as control [24]. MICs to nalidixic acid were also measured in the presence of efflux pump inhibitor phe-arg β-naphthylamide.

### 2.3. Mutational analysis of the quinolone-resistance determining regions of *gyrA *and *parC*

DNA was extracted from each quinolone-resistant isolate, using the Promega Wizard genomic extraction kit. The quinolone-resistance determining regions of the *gyrA *and *parC *genes were amplified from DNA templates by PCR using Platinum PCR supermix (Invitrogen) and the primer pairs listed in Additional file 1, Table S1. PCR reactions began with a two-minute hot start at 94°C followed by 30 cycles of 94°C for 30s, annealing temperature, 30s and 72°C for 30s. *gyrA *amplifications were annealed at 58°C and *parC *reactions were annealed at 52°C. *E. coli *K-12 MG1655 [25] was used as a control. Amplicons were sequenced on both strands and predicted peptide sequences were compared to the corresponding gene from the MG1655 genome [25] by pair-wise FASTA alignments.

### 2.4. Identification of horizontally-acquired quinolone-resistance genes

*qnrA*, *qnrB*, and *qnrS *were identified with PCR using the primer pairs published by Wu et al [26]. The primers of Liu et al [27] were used to screen for the *qepA *gene (Additional file 1, Table S1). Amplicons were sequence-verified.

### 2.5. Organic solvent tolerance

The qualitative organic solvent tolerance test used by Wang et al [18] was employed. Briefly 10^3^-10^4 ^mid-log phase bacteria were spotted onto glass plates of LB agar and overlaid with hexane, cyclohexane or a 1:1 [vol:vol] mixtures of these solvents. Strains were scored as producing no growth under any solvent (-), growth under all solvents (+++) or some solvents (+ or ++). *Pseudomonas aeruginosa *strain PA14 and *E. coli *MG1655 were used as positive and negative controls respectively.

### 2.6. Determination of transcript levels by quantitative real-time PCR

Total RNA was extracted from late log-phase cultures using the Qiagen RNeasy Protect bacterial minikit. Following reverse transcription using the Taqman Reverse Transcription Reagents and random hexamers (Applied Biosystems), quantitative real-time PCR with SYBR green was performed using primer pairs specific for genes of the components of the efflux pumps or efflux pump regulators using primers of Nishino et al. [28] and Bohnert *et al*. [29] (Additional file 2, Table S2). The reactions were run on the StepOne Real-time PCR system and performed according to manufacturers recommendations (Applied Biosystems). Gene expression levels were compared to the control strain MG1655, and analyzed using the 2^-ΔΔCt ^method [30]. The *rrsA *gene was used as the normalizing gene. Each reaction was performed in quadruplicate.

### 2.7. Flagellin typing

*fliC *PCR-restriction fragment length polymorphism (RFLP) typing was performed as described by Fields *et al *[31]. The *fliC *gene of test *E. coli *strains was amplified using the primers F-FLIC1 and R-FLIC2 (Additional file 1, Table S1). Amplicons were digested with *Rsa*I and restriction profiles were discriminated after electrophoresis on 2.5% agarose gels.

### 2.8. Multi-locus sequence typing

PCR primers listed in Additional file 1, Table S1 were used to amplify gene fragments from the *adk, fumC, gyrB, icd, mdh, purA *and *recA*, as described by Wirth et al [32]. Amplified DNA products were sequenced from both ends. Allele assignments were made at the publicly accessible *E. coli *MLST database accessible from http://www.mlst.net/. Phylogenetic inferences about ancestral allelic profiles and strain interrelatedness were made using eBURSTv3 at http://eburst.mlst.net/ defining sequence type (ST) complexes, that is clonal complexes, based on groups sharing six identical alleles.

### 2.9. Prescription audit of antimicrobial use

Prescription sheets were obtained with permission from the Obafemi Awolowo University Ile- Ife, Nigeria for 1984, 1985 and 1986; for 1994, 1995 and 1996 and for 2004, 2005 and 2006. Data from the prescription sheets was collated into a spreadsheet anonymously and then the prescription sheets were returned to the University Health Centre. For each year that was evaluated, 300 prescriptions were randomly selected for each month, permitting evaluation of 4-8% of the prescriptions that were actually filled. Prescriptions for all oral and topical antimicrobials, filled at the institution's Pharmacy were documented and units of measurement were standardized to daily-defined doses were computed using the AB Consumption Calculator tool [33].

## 3. Results

### 3.1. Emergence of fluoroquinolone resistance among commensal *E. coli *from south-western Nigeria

Our previous studies which tracked resistance in fecal *E. coli *isolates between 1986 and 1998 found uncommon (0-3.2%) and, in all but one case, low-level resistance to nalidixic acid and no fluoroquinolone resistance [21]. All but one of the nalidixic acid-resistant isolates identified before 1998 produced an inhibition zone diameter of at least 10 mm (Nalidixic acid sensitive strains have zone diameters > 18 mm. Resistant strains have zone diameters < 14 mm). Due to repeated power cuts in Nigeria, that strain archive is lost and none of those isolates are available for analysis. Employing an identical protocol in 2005 we recovered nalidixic acid non-susceptible *E. coli *from 18 (21.7%) volunteers. Four isolates exhibited high-level nalidixic acid resistance, producing no inhibition zone and MICs of ≥ 4 μg ml^-1^, and one of these was fluoroquinolone-resistant (Table 1). The remaining isolates exhibited low-level nalidixic acid resistance similar to that seen with earlier isolates reported previously [21]. No 2005 isolate carried horizontally-transmitted *qnr *or *qepA *genes but the four high-level nalidixic acid-resistant strains had mutations in the quinolone-resistance determining regions (QRDR) of quinolone target *gyrA *and the single FQREC isolate had an S80I substitution in ParC (Table 1).

**Table 1:** Quinolone-specific resistance mechanisms in 2005 and 2009 nalidixic acid-resistant isolates

Number of isolates	Nalidixic acid MIC	Ciprofloxcin	GyrA SNPs	ParC SNPs	Horizontally-acquired
2005					

3	8-32	Sensitive	S83L	-	-

1	1024	Resistant	S83L, D87N	S80I	-

2009					

2	256	Sensitive	D101G*	-	-

1	16	Sensitive	S83L	-	-

1	1024	Resistant	S83L, D87N	-	-

4	> 1024	Resistant	S83L, D87N	S80I	-

1	64	Sensitive	-	S94F*	-

1	256	Sensitive	-	V54I*	-

1	32	Resistant	-	Y74C*	*qnrS1*

5	16-256	Resistant	-	-	*qnrS1*

*Previously unreported SNP for which there is no evidence of functional contribution to resistance

In 2009, we recovered nalidixic acid-resistant *E. coli *from 35 (34.5%) volunteers of which 11 isolates were fluoroquinolone non-susceptible. Isolates obtained from nine individuals in 2009 harbored one or more non-synonymous mutations in *gyrA *and *parC *QRDRs and six isolates from the same year carried the horizontally-transmitted quinolone-resistance gene *qnrS1 *[34] (Table 1). Altogether, quinolone-specific mechanisms of resistance were identified in 16 isolates from 15 (17.4%) individuals in 2009, compared to 4 (4.8%) in 2005 (p < 0.05, Chitest with Yates correction for small numbers). All FQREC strains carried two or more QRDR mutations and/or *qnrS1 *(Table 1).

### 3.2. Molecular basis for nalidixic acid resistance in isolates obtained in 2005, during the transition to fluoroquinolone resistance

We isolated 21 nalidixic-acid resistant *E. coli *strains from 18 volunteers in Nigeria in 2005 and one of these was resistant to the fluoroquinolone ciprofloxacin. The majority (17) showed low-level nalidixic acid resistance and did not have mutations in *gyrA *or *parC*, nor did they carry horizontally-transmitted quinolone resistance *qnr *and *qepA *genes. Most of these isolates showed undue tolerance to organic solvents, suggesting that overactive efflux pumps may account for resistance (Table 2). We measured nalidixic acid MICs by agar dilution in the absence and presence of the RND-type efflux pump inhibitor phe-arg β-naphthylamide. As shown in Table 2 test strains showed an 8-fold or greater reduction in the concentration of nalidixic acid needed to inhibit growth in the presence of phe-arg β-naphthylamide, strongly suggesting a role for up-regulated efflux pumps. To confirm efflux pump upregulation, we used quantitative real-time PCR (qPCR) to measure transcription of 13 genes representing the major known efflux systems in *E. coli*. Compared to *E. coli *K-12 strain MG1655, sixteen isolates showed a two-fold or greater upregulation in transcription of the outer-membrane extrusion factor gene *tolC*. Seventeen of the isolates showed two-fold or greater increases in transcription of one or more other efflux genes and at least half of the targets were upregulated in most isolates (Table 2). The *acrA *efflux pump gene and *acrR*, encoding the gene's transcriptional regulator, and their promoters, were sequenced from five strains but none contained nucleotide polymorphisms that could account for increased efflux. We next examined transcription of six genes encoding regulators of efflux genes, including *acrR*, by qPCR and most strains showed upregulated transcription of one or more of these genes (Table 2). Of the three *E. coli *strains we employed as controls, strains ATCC 25922 and MC4100 produced fold changes for all genes ranging between 0.5 and 1.4 (compared to K-12 strain MG1655) for the different genes but we saw a two-fold increase for NCTC 10418 for nine efflux pump genes but none of the efflux regulators. Twelve of the QREC from Nigeria showed a four-fold or greater fold change in transcription of at least one gene encoding an efflux pump component and all but one of these strains had a two-fold of greater fold upregulation of one or more regulators. Thus, elevated efflux is the most likely explanation for low-level resistance seen in most of the 2005 isolates.

**Table 2:** Multilocus sequence types and antimicrobial efflux mechanisms in 2005 quinolone-resistant isolates

QREC Strain	MLST ST	ST Complex	fliC type	Resistance Profile	Nalidixic acid MIC (μg/ml)	Organic solvent tolerance	Nalidixic acid fold inhibition with PAβN	Δ GyrA	Δ ParC	Transcription fold change-tolC	Efflux pump genes with transcription fold change ≥ 4	Efflux regulator genes with transcription fold change 2-8	Efflux regulator genes with transcription fold change > 8
01A	10	10	A	A NSLTR	32	+	4	S83L	-	2.0	*emrK*	*-*	*rob*

05C	10	10	B	NS TR	4	++	8	-	-	1.0	*mdtA*	*-*	*-*

23A	10	10	B	AC NSLTR	4	++	8	-	-	2.0	*mdtA*	*marA, soxS, beaS, marR, acrR*	*-*

33A	10	10	B	AC NSLTR	4	++	8	-	-	4.5	*acrA, acrD, acrE, bcr, cusB, emrA, emrD, emrK, fsr*,	*marA, soxS, evgA, marR, acrR*	*rob, baeS*

25A	10	10	D	A NSLTR	4	++	8	-	-	16.8	*acrA, acrD, acrE, bcr, cusB, emrA,emrD, emrK, fsr, macA, mdfA, mdtA*	*evgA*	*marA, soxS, rob, beaS, marR, acrR*

40B	10	10	E	N	4	+++	8	-	-	1.0	*-*	*-*	*-*

16A	34	10	E	NS	4	+++	16	-	-	4.2	*acrA, acrD, acrE, bcr, cusB, fsr, mdtA*	*marA, soxS, rob, marR, acrR*	*baeS*

49A	43	10	F	N	4	+	8	S83L	-	2.2	*-*	*-*	*-*

23B	10	10	C	C N LTR	4		8	-	-	2.2	*-*	*-*	*-*

31A	494	none	C	A NSLTR	4	++	8	-	-	2.2	*acrD, acrE, bcr, emrA, mdtA*	*baeS*	*-*

38C	521	none	C	NS R	4	++	8	-	-	4.3	*acrA, acrD, acrE, bcr, cusB, emrA, emrD, fsr, mdfA, mdtA*	*marA, soxS, evgA, rob, marR, acrR*	*beaS*,

22A	452	none	C	NSLTR	4	+	8	-	-	1.1	*-*	*-*	*-*

31B	452	none	C	NSLTR	4	-	4	-	-	2.5	*-*	*-*	*-*

33B	504	none	K	NSLT	1024	+++	16	S83L	-	1.0	*-*	*-*	*-*

34B	498	467	G	NS	4	++	8	-	-	2.1	*-*	*baeS*	*-*

32C	503	13	H	NSLTR	4	++	8	-	-	1.0	*-*	*baeS*	*-*

30A	156	156	I	AC NSLTR	4	++	16	-	-	4.3	*acrA, acrD, cusB, emrA, emrK, mdfA*	*soxS, baeS*	*-*

27A	448	448	J	ACPNSLTR	1024	+++	2	S83L, D87N	S80I	33.2	*acrA, acrD, acrE, bcr, cusB, emrA, emrD, fsr, macA, mdfA, mdtA*	*-*	*marA, soxS, evgA, rob, beaS, marR, acrR*

09C	517	none	L	AC NSLTR	4	++	8	-	-	8.6	*acrA, acrD, acrE, bcr, cusB, emrA,emrD, emrK, fsr, mdfA, mdtA*	*marA, soxS, evgA, rob, marR, acrR*	*beaS*

38A	517	none	N	A NSLTR	4	++	8	-	-	4.2	*acrR, acrD, acre, bcr, cusB, fsr, mdtA*	*marA, soxS, evgA, rob, baeS, marR, acrR*	

ATCC 25922	ND	ND	ND	-	2	-	8	ND	ND	0.4	*-*	*-*	*-*

NCTC10418	ND	ND	ND	-	2	-	4	ND	ND	2.1	*-*	*-*	*-*

ND: Not Determined

Antimicrobials: A, ampicillin; C, chloramphenicol; P, ciprofloxacin; N, nalidixic acid; S, streptomycin; L, sulfonamides; T, tetracycline; R, trimethoprim

MLST of quinolone-resistant *E. coli *revealed a startling lack of diversity with the 21 strains representing only 13 sequence types. Nine isolates belonging to sequence type (ST) complex 10 and seven specifically belonging to ST 10. ST452 (2 isolates) and its single locus variant ST521, represented a second frequently occurring clonal complex and we identified one ST156 strain, which along with ST10 has recently been identified among quinolone-resistant *E. coli *in Ghana (Table 2)[35].

### 3.3. Antimicrobial prescription trends at the University Health center

As shown in Table 3, prescribing practices at the Health Center were highly physician-specific in the 1980s but by 2004-2006, had become more uniform. However, with the increasing uniformity, we observed an overall increase in the mean and median number of drugs per prescription (Table 3). While only one of five physicians averaged more than four drugs per prescription in 1984-1986, in 2004-2006, seven of nine physicians were prescribing an average of four drugs or more per patient. In spite of the very small number of prescribing physicians, this difference approached statistical significance (p = 0.06 Fishers exact test). Anti-infectives, the most commonly prescribed medicines at the health center, represented 49.3% of the drugs listed on prescriptions between 1984 and 1986, 21.9% in 1994-1996 and 38.7% in 2004-2006.

**Table 3:** Prescribing patterns of physicians in the University Health Center

	1984-1986	1994-1996	2004-2006
Number of prescribing physicians	7	10	9

Mean ± SD drugs per prescription	3.1 ± 1.1	3.2 ± 0.5	4.5 ± 0.5

Median (Range for different physicians) drugs per prescription	3.1 (1.6-5)	3.25 (2.5-4)	4.71 (3.5-4.9)

**Figure 1:**
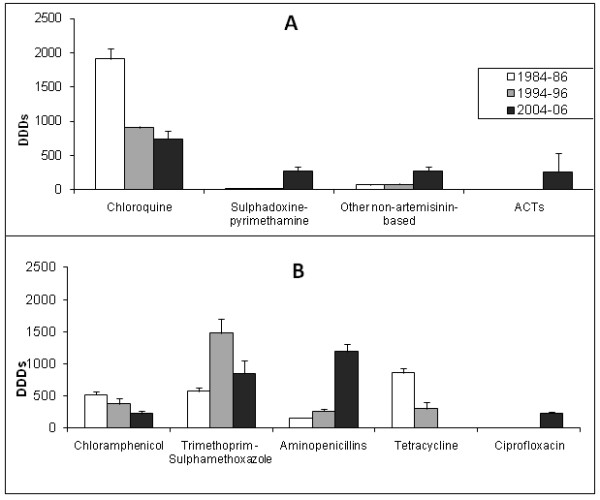
**Antibacterial and Antimalarial prescription at the University Health Center**. Daily defined doses (DDDs) of antimalarials (A) and antibacterials (B) dispensed from the Health center pharmacy in the 1980s, 1990s and 2000s.

In the 1980s, chloroquine accounted for over 96% of all antimalarials prescribed. Pyrimethamine alone was prescribed in prophylactic doses only and sulphadoxine-pyrimethamine, which is more expensive than chloroquine but much cheaper than other antimalarials, was rarely prescribed during the first decade. In the second decade of the study, this drug combination was prescribed for 5% of patients receiving an antimalarial and by the 2000s, it was recommended for almost 40% (Figure 1). With the exception of Artemisinin Combination Therapies (ACTs), use of which increased considerably between 2004 and 2006, there were no significant within-decade differences in prescription rates for each antimalarial. However, antimalarial prescription choices changed considerably from one decade to the next. Discounting combinations, the greatest diversity of antimalarial drugs was prescribed in the middle decade, 1990s (Figure 1) and may have been prompted by emerging resistance and the absence of a set antimalarial policy. Amodiaquine and halofantrine represented a significant proportion of prescriptions in the 1990s, but by the 2000s, all patients not receiving chloroquine or amodiaquine received ACTs [36]. A decline in chloroquine prescription from 45.4% to 25.1% occurred in parallel with ACT roll out. However chloroquine prescription was never discontinued and it remained the antimalarial of choice for most patients with clinical malaria throughout the duration of the study (Figure 1).

Like antimalarial prescription, antibacterial prescription altered significantly over the study period (Figure 1). Chloramphenicol and tetracycline, drugs of choice in the 1980s, were prescribed less in the following decades as drugs that did not have toxicity concerns became available and more affordable. Prescription of the aminopenicillins ampicillin and amoxicillin for example increased eight-fold from between the first and last decade. As with antimalarials, with the exception of tetracycline, drugs that fell out of favor were never completely discontinued so that selective pressure from them was never removed. Ciprofloxacin was the only quionolone prescribed at the health center and was introduced in the 2000s, the decade in which *E. coli *with quinolone-specific resistance mechanisms were detected.

## 4. Discussion

This study allowed us to broach the almost untestable question of population-level selective forces for quinolone resistance in bacteria within a malaria-endemic area. The long duration of resistance monitoring offered significant advantages in spite of unavoidable limitations. We deemed it important to replicate the methodology of earlier years, which means that we only examined a small sample of the commensal *E. coli *population carried by each volunteer. Other limitations of this study include the fact that our prescription audit did not sample the complete catalogue of selective pressure for resistance. It is unlikely that patients who receive free medications at the health center would have chosen to purchase their prescriptions elsewhere, however, we cannot rule out the possibility that patients may have purchased other medicines to augment, or replace, those offered at the health center. Prescriptions for parenteral drugs are handled differently and records are not retained long-term at the center and therefore injectables were not evaluated, even though it is well known that many commonly used antimicrobials are administered intramuscularly in Nigeria [37].

The quinolone resistance rates and their association with access to quinolones that we have recorded in this study compare with those from reports from other parts of Nigeria and West Africa, and all support the hypothesis that fluoroquinolone introduction immediately precedes the evolution of FQREC [38-42]. However, it is difficult to draw even cautious conclusions across multiple studies and or from anecdotes. Therefore, in spite of the aforementioned limitations, the present study provides coherent evidence to support the hypothesis because it tracks resistance and use in a select area using simple but reproducible methodology. Our data show that quinolone resistance emerged in commensal *E. coli *after the 1990s in Ile-Ife, Nigeria, and that our 2005 isolate collection, largely but not entirely comprised of low-level resistors, appears to have been procured at a critical juncture-shortly after the evolution of resistance. By 2009, 16 (46%) of quinolone-resistant isolates showed high-level quinolone resistance, and resistance to the fluoroquinolones, attributable to quinolone-specific resistance mechanisms that have commonly been reported in other studies. Resistance-conferring mutations in *gyrA *and *parC *were the most commonly recorded of these resistance mechanisms. When horizontally-transmitted resistance evolved, *qnrS1 *was the most frequently detected transmissible allele among commensal *E. coli *from Vietnam and Ghana, where like Nigeria antimicrobials are freely available and fecal-oral transmission of bacteria is common [35,43]. Altogether, we observed rapid, stepwise evolution of FQREC between 2005 and 2009, from a very low baseline.

The predominance of ST10 among 2005 quinolone resistant isolates may be due to the overall predominance of ECOR phylogenetic group A strains in West Africa [44]. However, we have recently shown that this lineage is overrepresented among quinolone-resistant strains, as compared to sensitive strains in Ghana [35]. We performed in-depth analyses of the 2005 isolates from this study to get a sense of how resistance might have evolved and our data suggest that strains with overactive efflux may have been the predecessors of strains exhibiting quinolone-specific resistance mechanisms. The differences in efflux phenotypes, efflux genes transcription profiles and flagellin types among most of the strains belonging to the same ST suggest that the low-level resistance we detect is not primarily due to expansion of recently derived clones. Instead, the data appear to suggest that elevated efflux is more likely to be seen in specific lineages, likely those with overactive efflux pumps. This in turn points to the idea that certain lineages may be primed to evolve more stable, less costly resistance. Horizontally-transmitted *qnr *genes, including *qnrS1 *identified in this study have recently been implicated in providing low-level quinolone resistance that could provide strains with the opportunity to acquire subsequent mutations in quinolone targets and therefore higher-level stable resistance [45]. It is therefore worrisome that *qnrS1 *can now be commonly detected among *E. coli *commensals in Nigeria. More studies are needed to improve our understanding of the evolution and dissemination of quinolone resistance. We suggest that on-going and proposed studies tracking resistance across Africa include a focus on quinolone resistance at genetic as well as phenotypic levels and that more work is done to elucidate the genetic context of successful horizontally transmitted quinolone resistance genes as they appear in African countries, similar to work that has been done in Vietnam [43].

**Figure 2:**
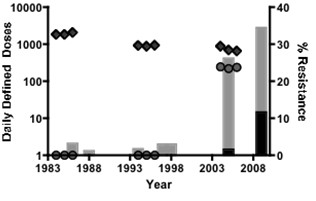
**Proportion of *Escherichia coli *isolates from students in Ile-Ife, Nigeria that were quinolone resistant (1986-2009, right axis) with grey portions of the bars indicating strains resistant to nalidixic acid alone and black shading representing strains resistant to nalixic acid and the fluoroquinolone ciprofloxacin**. Daily defined doses of chloroquine (diamonds) and ciprofloxacin (circles) dispensed from the University Health center pharmacy in 1984-86, 1994-96 and 2004-06 are plotted from the left axis.

Rampant non-prescription use, informal supply chains and a dearth of archival records make it challenging to quantify drug use in Nigeria. However, the health center at the University where we tracked resistance offers students and staff free health care and archives dispensed prescriptions. This long-term study was thus conducted in a setting that provided the rare opportunity to retrospectively study antimicrobial prescription patterns in an equatorial Africa locality where resistance was also tracked. We find that chloroquine prescription was inordinately high in the 1980s with 97% of patients visiting the health center receiving this drug. Chloroquine prescription declined steadily thereafter but never dropped below 500 daily-defined doses, with 25% of patients receiving this drug in 2006 (Figure 2). In contrast to the earlier report from Guyana [4], we do not find evidence of selection for FQREC from heavy and long-running chloroquine use in this setting. By contrast, quinolone antibacterials were never used before the 2000s and introduction of ciprofloxacin is temporally associated with evolution, and rising rates, of FQREC carriage by campus undergraduates. The significance of our findings cannot be over-emphasized since current efforts to 'bring back' chloroquine, an extremely effective and cheap antimalalarial, would be de-prioritized if there were adverse consequences on resistance in other organisms.

The number of drugs prescribed per patient and the common use of antimicrobial drugs we documented compare with studies performed elsewhere in Nigeria for the respective periods [46,47]. Overall antimicrobial use was characterized by heavy dependence on three drug classes or less and a shift in favored drug categories from one decade to another, without withdrawal of earlier favorites. This drug use pattern, typified by the antimalarial chloroquine, favors the emergence of stable resistance [40,48,49].

Quinolones are drugs of last resort that are increasingly employed in Nigeria and other African countries to manage common bacterial infections. In the face of high and rising resistance to more affordable antimicrobials and the unavailability of newer, patent protected antibacterials, there is currently no real alternative to their use in West Africa. Evolution of resistance to the quinolones among *E. coli *has important clinical implications because resistance patterns in commensals typically mirror those seen in pathogens exposed to similar selection. A recent report from neighboring Cameroon suggests that quinolone resistance has emerged among *Salmonella enterica *serovar Typhi there [50] and we have reported quinolone resistance in epidemic *Vibrio cholerae *from Ghana [51].

## Conclusion

This study charts the appearance of quinolone-resistant E. coli in a Nigerian malaria endemic community, which has seen heavy and long-term use of chloroquine. The study demonstrates that quinolone resistance was not temporally associated with chlrooquine use but appeared shortly after fluoroquinolone introduction. The diversity of quinolone-specific resistance mechanisms that emerged following ciprofloxacin introduction points to an urgent need for therapeutic alternatives and resistance containment. Our findings suggest that the useful lifespan for newly introduced antimicrobials that have been used extensively elsewhere can be short. High prices of patent-protected drugs delay their introduction to developing countries and strategies for ensuring early but controlled access to novel antimicrobials may be necessary to ensure medium- to long-term efficacy in resource-limited settings.

## Competing interests

INO is an editor of BMC Infectious Diseases. No other competing interests are declared by all authors.

## Authors' contributions

AL co-conceived the study, collected isolates and collected antimicrobial use data. JLC designed and performed molecular experiments, analysed and interpreted data. RSL performed molecular experiments, analysed and interpreted data, and contributed to writing the paper. BWO and AOA collected isolates and performed microbiology experiments. JW designed experiments and contributed to writing the paper. INO co-conceived the study, performed microbiology and molecular experiments, analysed and interpreted data and wrote the manuscript. All authors read and approved the final manuscript.

## Pre-publication history

The pre-publication history for this paper can be accessed here:

http://www.biomedcentral.com/1471-2334/11/312/prepub

## Supplementary Material

Additional file 1 **Table S1: **Oligonucleotide primers used for standard PCR reactions. Click here for file

Additional file 2 **Table S2: **qPCR primers. Click here for file
