# Supplementary material for: Rapid evolution of fluoroquinolone-resistant Escherichia coli in Nigeria is temporally associated with fluoroquinolone use
Source: BMC Infect Dis. 2011 Nov 7;11:312. doi: 10.1186/1471-2334-11-312 (PMC3226678; doi:10.1186/1471-2334-11-312)
Supplement: Additional file 1 — Table S1: Oligonucleotide primers used for standard PCR reactions. [file 1471-2334-11-312-S1.DOC]

**Additional file 1**

**Table S1:** Oligonucleotide primers used for standard PCR reactions

| **Target gene** | **Primer** | **Primer Sequence** | **Purpose** | **Refer-ence** |
| --- | --- | --- | --- | --- |
| *gyrA* | gyrA12004 | TGC CAG ATG TCC GAG AT | *gyrA* QRDR amplification | (1) |
|  | gyrA11753 | GTA TAA CGC ATT GCC GC |  |  |
| *parC* | EC-PAR-A | CTG AAT GCC AGC GCC AAA TT | *parC* QRDR amplification | (2) |
|  | EC-PAR-B | GCG AAC GAT TTC GGA TCG TC |  |  |
| *qnrA* | qnrA-1A | TTC AGC AAG ATT TCT CA | *qnrA* detection | (3) |
|  | qnrA-1B | GGC AGC ACT ATT ACT CCC AA |  |  |
| *qnrB* | qnrB-CS-1A | CCT GAG CGG CAC TGA ATT TAT | *qnrB* detection | (3) |
|  | qnrB-CS-1B | GTT TGC TGC TCG CCA GTC GA |  |  |
| *qnrS* | qnrS-1A | CAA TCA TAC ATA TCG GCA CC | *qnrS* detection | (3) |
|  | qnr-1B | TCA GGA TAA ACA ACA ATA CCC |  |  |
| *qepA* | qepA-F | GCAGGTC CAGCAGCGGGTAG | *qepA* detection | (4) |
|  | qepA-R | CTTCCTGCCCGAGTATC GTG |  |  |
| *adk* | *adk*F | ATTCTGCTTGGCGCTCCGGG | MLST | (5) |
|  | *adk*R | CCGTCAACTTTCGCGTATTT |  |  |
| *fumC* | *fumC*F | TCACAGGTCGCCAGCGCTTC | MLST | (5) |
|  | *fumC*R | GTACGCAGCGAAAAAGATTC |  |  |
| *gyrB* | *gyrB*F | TCGGCGACACGGATGACGGC | MLST | (5) |
|  | *gyrB*R | ATCAGGCCTTCACGCGCATC |  |  |
| *icd* | *icd*F | ATGGAAAGTAAAGTAGTTGTTCCGGCACA | MLST | (5) |
|  | *icd*R | GGACGCAGCAGGATCTGTT |  |  |

| *mdh* | *mdh*F | ATGAAAGTCGCAGTCCTCGGCGCTGCTGGCGG | MLST | (5) |
| --- | --- | --- | --- | --- |
|  | *mdh*R | TTAACGAACTCCTGCCCCAGAGCGATATCTTTCTT |  |  |
| *purA* | *purA*F | CGCGCTGATGAAAGAGATGA | MLST | (5) |
|  | *purA*R | CATACGGTAAGCCACGCAGA |  |  |
| *recA* | *recA*F | CGCATTCGCTTTACCCTGACC | MLST | (5) |
|  | *recA*R | TCGTCGAAATCTACGGACCGGA |  |  |

**References**

1. Wang H, Dzink-Fox JL, Chen M, & Levy SB (2001) Genetic characterization of highly fluoroquinolone-resistant clinical *Escherichia coli* strains from China: role of *acrR* mutations. *Antimicrob Agents Chemother* 45(5):1515-1521.

2. Deguchi T*, et al.* (1997) Detection of mutations in the *gyrA* and *parC* genes in quinolone-resistant clinical isolates of *Enterobacter cloacae*. *J Antimicrob Chemother* 40(4):543-549.

3. Wu J-J, Ko W-C, Tsai S-H, & Yan J-J (2007) Prevalence of plasmid-mediated quinolone resistance determinants QnrA, QnrB, and QnrS among clinical isolates of *Enterobacter cloacae* in a Taiwanese hospital. *Antimicrob. Agents Chemother.* 51(4):1223-1227.

4. Liu J-H*, et al.* (2008) Coprevalence of plasmid-mediated quinolone resistance determinants QepA, Qnr, and AAC(6')-Ib-cr among 16S rRNA methylase RmtB-producing *Escherichia coli* isolates from pigs. *Antimicrob. Agents Chemother.* 52(8):2992-2993.

5. Wirth T*, et al.* (2006) Sex and virulence in *Escherichia coli:* an evolutionary perspective. *Mol Microbiol* 60(5):1136-1151.
