# Supplementary material for: Rapid evolution of fluoroquinolone-resistant Escherichia coli in Nigeria is temporally associated with fluoroquinolone use
Source: BMC Infect Dis. 2011 Nov 7;11:312. doi: 10.1186/1471-2334-11-312 (PMC3226678; doi:10.1186/1471-2334-11-312)
Supplement: Additional file 2 — Table S2: qPCR primers. [file 1471-2334-11-312-S2.DOC]

**Additional file 2**

**Table S2:** qPCR primers

| **Gene** | **Forward or Reverse Primer?** | **Sequence (5' to 3')** | **Position in MG1655 genome (Genbank Number: U00096)** | **Amplicon Size**  **(bp)** | **Reference** |
| --- | --- | --- | --- | --- | --- |
| *rrsA* | F | CGGTGGAGCATGTGGTTTAA | 2728190 | 29 | (1) |
| *rrsA* | R | GAAAACTTCCGTGGATGTCAAGA | 2728219 | 29 | (1) |
| *acrA* | F | GTCTATCACCCTACGCGCTATCTT | 484005 | 29 | (1) |
| *acrA* | R | GCGCGCACGAACATACC | 483976 | 29 | (1) |
| *acrD* | F | GTACCCTGGCGATTTTTTCATT | 2585683 | 71 | (1) |
| *acrD* | R | CGGTCACTCGCACATTCG | 2585754 | 71 | (1) |
| *acre* | F | CGTGATTGCCGCAAAAGC | 3411966 | 69 | (1) |
| *acre* | R | TTGGCGCAGTGACTTTGGTA | 3412035 | 69 | (1) |
| *Bcr* | F | TGTTTTTCTGTTCGTGATGACCAT | 2276981 | 26 | (1) |
| *Bcr* | R | GGAACATATTTAACGCGCCAAT | 2276955 | 26 | (1) |
| *cusB* | F | CGCTTACCGTGGGCGATA | 597111 | 71 | (1) |
| *cusB* | R | TTCCACCCAGTCAGGAATGG | 597182 | 71 | (1) |
| *emrA* | F | GCGAATATTGAGGTGCAGAAAA | 2809827 | 64 | (1) |
| *emrA* | R | GGCACACGGCGGTTGTA | 2809891 | 64 | (1) |
| *emrD* | F | GTGGATCCCCGACTGGTTT | 3852412 | 79 | (1) |
| *emrD* | R | CCCGGCACCGAAAAAGA | 3852491 | 79 | (1) |
| *emrE* | F | GGTATTGTCCTGATTAGCTTACTGTCAT | 567736 | 89 | (1) |
| *emrE* | R | GCACAAATCAACATCATGCCTATAA | 567825 | 89 | (1) |
| *emrK* | F | GCGCTTAAACGTACGGATATTAAGA | 2480723 | 33 | (1) |
| *emrK* | R | ACTGTTTCGCCGACCTGAAC | 2480690 | 33 | (1) |
| *Fsr* | F | TGGTGTTGGCGCAAATCA | 503329 | 36 | (1) |
| *Fsr* | R | TCGTCGCTTTGGGTTTTCC | 503293 | 36 | (1) |
| *macA* | F | CGGTGATTGCCGCACAA | 919069 | 64 | (1) |
| *macA* | R | TTACCAGCATGGCGCTCAT | 919133 | 64 | (1) |
| *mdfA* | F | CTTGCTGTTAGCGCGTCTGA | 883712 | 63 | (1) |
| *mdfA* | R | GCCAGCCGCCCATAATAAT | 883775 | 63 | (1) |
| *mdtA* | F | CGCCGTAGAACAGGCAGTTC | 2152235 | 76 | (1) |
| *mdtA* | R | TGCGCACCGTAACGGTATTA | 2152311 | 76 | (1) |
| *tolC* | F | CCGGGATTTCTGACACCTCTT | 3176957 | 88 | (1) |
| *tolC* | R | TTTGTTCTGGCCCATATTGCT | 3177045 | 88 | (1) |
| *marA* | F | AAACCGGTCATTCATTAGGC | 1617743 | 175 | (2) |
| *marA* | R | GTATTTATGCGGCGGAACAT | 1617918 | 175 | (2) |
| *soxS* | F | ACCAGCCGCTTAACATTGAT | 4274889 | 154 | (2) |
| *soxS* | R | ACATAACCCAGGTCCATTGC | 4274735 | 154 | (2) |
| *evgA* | F | TTCTTGTTTCGATAAGGAGTCGAGTT | 2482389 | 38 | (2) |
| *evgA* | R | TGCTATTTCCCCTTCTCTCTCAAC | 2482146 | 38 | (2) |
| *Rob* | F | CCCAGAGCTACTCCTGTTCG | 4632437 | 240 | (2) |
| *Rob* | R | CGGTTCCCAGACCTTCATAG | 4632197 | 240 | (2) |
| *baeSq* | F | TGGTCAACGGTGCGGAAGT | 2161298 | 79 | This Study |
| *baeSq* | R | ATCGAAATTGATATCAGTATTGC | 2161377 | 79 | This Study |
| *marRq* | F | CTGCTTAACGAGTATCTGTCT | 1617226 | 65 | This Study |
| *marRq* | R | GGATAGAGCAGAGCACCTTA | 1617291 | 65 | This Study |
| *acrRq* | F | TTCCACAAATGCGAATTTGTCG | 485325 | 70 | This Study |
| *acrRq* | R | ACTTTCCAGACAGAGATTACGT | 485395 | 70 | This Study |

**References**

1. Nishino K, Senda Y, & Yamaguchi A (2008) CRP regulator modulates multidrug resistance of *Escherichia coli* by repressing the *mdtEF* multidrug efflux genes. *J Antibiot (Tokyo)* 61(3):120-127.

2. Bohnert JA, Schuster S, Fahnrich E, Trittler R, & Kern WV (2007) Altered spectrum of multidrug resistance associated with a single point mutation in the *Escherichia coli* RND-type MDR efflux pump YhiV (MdtF). *J Antimicrob Chemother* 59(6):1216-1222.
